# Phenolic Iron Complexes Protect Glacier Ice Algae (Zygnematophyceae) Against Excessive UV and VIS Irradiation

**DOI:** 10.1111/1758-2229.70149

**Published:** 2025-07-14

**Authors:** Lenka Procházková, Peter Mojzeš, Jan Ráček, Linda Nedbalová, Daniel Remias

**Affiliations:** ^1^ Department of Ecology Faculty of Science, Charles University Prague Czech Republic; ^2^ Institute of Physics Faculty of Mathematics and Physics, Charles University Prague Czech Republic; ^3^ Department of Botany Faculty of Science, Charles University Prague Czech Republic; ^4^ Institute of Microbiology Czech Academy of Sciences Prague Czech Republic; ^5^ Department of Environment & Biodiversity University of Salzburg Salzburg Austria

**Keywords:** cryoflora, glaciers, polyphenols, Raman microscopy, secondary pigmentation

## Abstract

Melting glacier surfaces are increasingly affected by blooms of psychrophilic microalgae, which darken the ice and lower its albedo, accelerating melting. These microalgae contain distinct vacuoles filled with brownish pigments that were earlier described as the unusual plant phenol purpurogallin. Recently, we discovered so far unreported, large amounts of iron dissolved in aqueous extracts of the glacier ice algae *Ancylonema alaskanum*. Since the vacuole content was very dark but the chromatographically isolated, aforementioned phenol was only yellowish, a putative complexation of iron with purpurogallin was assumed to be the reason. Application of several protocols, including Raman microscopy on both living cells and extracts, provided strong evidence that this microalga sequesters iron and forms organic metal complexes. Consequently, substantial amounts of so far uncharacterised Fe‐complexes of purpurogallin are inferred to be present in *Ancylonema*, and that putative polymerisation of this compound impeded an earlier analytical discovery. This finding holds significant ecological implications for cold regions. The pigmentation not only enhances the tolerance of glacier ice algae to excessive UV and visible radiation but also influences our current understanding of the biochemical iron cycle in cryosphere‐dominated polar and alpine regions. Further downstream consequences of this biological iron source remain to be elucidated.

## Graphical abstract

We brought evidence that glacier ice alga *Ancylonema alaskanum* (Zygnematophceae) sequesters iron and forms organic metal complexes. We found that phenols were already present in the green *Ancylonema* *alaskanum* laboratory strain. Our study showed that their vacuole turned brownish due to enhanced iron‐phenol complexation under nutrient limitation with high light and UV. The novelty of this study is the discovery of iron‐purpurogallin complexes that enhance cellular tolerance to elevated ultraviolet radiation and visible light levels.

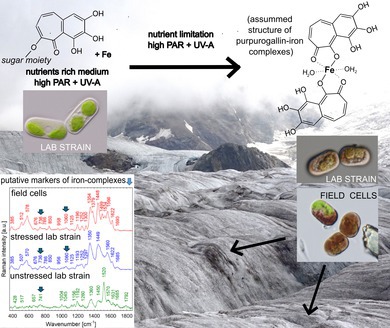

## 1 Introduction

Specialised photoautotrophic microbes thrive on bare glacier surfaces in alpine and polar regions (e.g., Millar et al. 2024; Onuma et al. 2023). Under favourable conditions, they cause “dark blooms”, detectable by satellite imagery, that dominate extensive parts of the western Greenland ice sheet (Williamson et al. 2018; Wang et al. 2018). This phenomenon is of global importance because it reduces the albedo of the ice, thereby accelerating ice melt and contributing to sea‐level rise (Williamson et al. 2020). The majority of these algae belong to the genus *Ancylonema* (Remias et al. 2023). These green algae are classified within the Zygnematophyceae, a group now recognised as the closest relatives to the land plants (Rensing 2020). Extreme abiotic conditions at the glacier surface include high levels of visible (VIS) and ultraviolet radiation (UV); the prevailing following values were reported during a sunny day from a Swiss glacier: 2113 μmol photons m^−2^ s^−1^ (PAR), 4.55 mW cm^−2^ (UV‐A) and 0.158 W m^−2^ (UV‐B), respectively (Procházková et al. 2021).

Secondary metabolites likely played a crucial role during the terrestrialisation of plants, enabling cells to adapt to changing environmental conditions, such as temperature fluctuation, excessive irradiance, and oxidative stress (Dadras et al. 2023). The flavonoids of land plants are a prominent example, acting as antioxidants, screening compounds, or repellents (Kumar et al. 2023). The Zygnematophyceae, as a sister group to land plants, are sometimes found in (semi‐)terrestrial habitats, including tree bark, mosses, wet rocks, or melting ice. Concurrently, a variety of abundant, vacuole‐bound phenols emerged in these derived green algae (Permann, Becker, and Holzinger 2022). Such phenols may either represent a form of ancestors to the true flavonoids or at least vicariously fulfil their ecological roles biochemically.

Characteristically, the vacuoles of glacier ice algae possess an abundant brownish secondary pigmentation. The pigment was previously identified as a phenol uncommon in the plant kingdom—a glycosylated purpurogallin derivative (Remias, Schwaiger, et al. 2012). It was proposed to protect DNA from the damaging effects of mutagenic UV radiation. Additionally, there is the issue of protecting chloroplasts from harmful levels of VIS irradiation. This protection is crucial because excessive VIS irradiation can inhibit the process of photosynthesis. Such concerns are particularly relevant for organisms living on exposed ice surfaces, where these environmental stressors are prevalent.

In other species of the Zygnematophyceae, such as *Zygnema* spp. (Pichrtová et al. 2013) or *Zygogonium ericetorum* (Newsome and van Breemen 2012), the ecophysiological role of phenols was acknowledged too. In the case of the latter, a purple vacuolar pigmentation was identified as a highly branched polymer of glucose containing traces of ester‐linked polyphenolic moieties such as gallic acid. The purple colour of the polysaccharide is due to the complexation of the polyphenolic groups by ferric iron (Newsome and van Breemen 2012). The occurrence of such unusual compounds in these algae, combined with our preliminary discovery of high iron concentrations in aqueous extracts of *Ancylonema alaskanum* from an alpine glacier, motivated us to investigate whether a similar complexation between phenols and iron exists in glacier ice algae.

Iron is an essential element for all living organisms, representing a redox cofactor in many metabolic pathways, especially the respiratory chain, thylakoid reactions (Raven et al. 1999), and nitrogen fixation (Whittaker et al. 2011) have high iron demand. As demonstrated by mesoscale experiments, adding iron to high‐nutrient–low‐chlorophyll regions in the ocean is sufficient to stimulate algal blooms (Boyd et al. 2007). Measurements of dissolved compounds are routinely made by filtration through 0.45 or 0.2 μm filters, and thus, these measurements are based on size, not speciation. Based on size fractionation, the dissolved Fe (< 0.2 or 0.45 μm) is recognised either as soluble Fe (< 0.02 μm) or colloidal/nanoparticulate Fe (0.02 to 0.2 or 0.45 μm). The ranges of soluble Fe concentrations in glacier ecosystems such as icebergs and glacial meltwater are similarly low—in 10s of nanomolar (Raiswell et al. 2018). The range of colloidal/nanoparticulate Fe concentrations in these habitats of icebergs and meltwater is higher: 100 or up to 1000 of nanomolar, respectively (Raiswell et al. 2018). While the total Fe concentration in glacier ice is generally low, the soluble Fe—though a small fraction—is crucial for microbial processes. Microbial iron uptake was evidenced in dense blooms of glacier ice algae on the western Greenland ice sheet (McCutcheon et al. 2021). The concentration of mineral iron at these bloom sites was lower when compared to dispersed cryoconite spots and clean ice sites. The measured concentration of dissolved iron in the dense blooms was two to four times higher (69 ± 20 μg·L^−1^) than in dispersed cryoconite spots (36 ± 5 μg·L^−1^) and clean ice sites (16 ± 13 μg·L^−1^) (McCutcheon et al. 2021).

In this study, we used the first known strain of a glacier ice alga (*Ancylonema alaskanum* CCCryo 565–23) whose cells remain green when grown at standard laboratory conditions, possessing unpigmented vacuoles (Remias and Procházková 2023). An exposure experiment was performed to induce dark pigment synthesis, and several analytical and observational techniques were implemented to test the presence of iron complexes, both in field and strain material. We believe that for certain microalgae, such an unusual vacuolar pigmentation characterised by broad UV and visible light absorption may play a similar ecophysiological role as anthocyanins—polyphenols derived from land plants.

## 2 Materials and Methods

### 2.1 Field Sampling and Identification

With the help of a field microscope (EM1, Archimedes Research Ltd.), a virtually monospecific bloom of *Ancylonema (A.) alaskanum* (sample WP274) was selected and harvested from surface glacier ice at Morteratsch Glacier, Switzerland (21 August 2021, GPS: N46 24.692 E9 56.085, altitude 2301 m a.s.l., Figure S1 online), as previously reported in Procházková et al. (2024). Light microscopy of sample WP274 confirmed the dominance of *A. alaskanum* , accompanied by scattered single cells or up to four‐celled filaments of *A. nordenskioeldii* (abundance ratio of the two species 20:1, Figure S2 online). Cells used for this study were kept illuminated at 5°C (16 h light: 8 h darkness at 50 μmol photons m^−2^ s^−1^) prior to further analyses. These fresh field cells were subjected to the micro‐Raman measurements 6 weeks after the sampling.

### 2.2 Exposure Experiments With Laboratory Strain

Strain CCCryo 565–23 of *Ancylonema alaskanum* (Zygnematophyceae, Streptophyta) (isolated from a sample collected in an Austrian glacier in 2020, Remias and Procházková 2023) was maintained at 5°C and 40 μmol photons m^−2^ s^−1^ in “Synthetic Freshwater Medium” (SFM; containing iron at 11.654 μmol L^−1^ FeCl_3_·6H_2_O; recipe see in the supplement of Remias and Procházková 2023). The laboratory medium such as SFM in its recipe approximates the dissolved Fe pool, using soluble Fe salts at concentrations comparable to or slightly higher than in situ dissolved Fe (0.2–9 μmol L^−1^ in glacial meltwater, Table 2 in Raiswell et al. 2018). In SFM, Fe is primarily in the soluble bioavailable form (chelated or free ions). In situ Fe occurs in both soluble and colloidal/nanoparticulate, the latter dominates (Raiswell et al. 2018), so Fe bioavailability is limited unless ligands or weathering occurs. This means that the Fe in glacier ice is mostly in particulate or complexed form, not purely aqueous, but environmental processes can alter its speciation and bioavailability. For the phenolic iron‐complex induction experiments, we selected one control medium and three stress media to increase the production of phenolics. These compounds are involved in the putative in vivo complexation with iron. The medium was modified either by omitting phosphorus and nitrogen sources (but still containing the iron source) or completely replacing it with deionised water or nutrient‐free medium with additional osmotic stress. In detail, the 250 mL acid‐washed glassware of Erlenmeyer flasks (*n* = 6) were inoculated with 100 mL of the following four media: (1) control conditions with eSFM (double nitrogen and phosphorus concentration compared to standard SFM); (2) deionised (Millipore) water and (3) SFM without nitrogen and phosphorus, (4) SFM without nitrogen and phosphorus but supplemented with additional NaCl (Table S1 online). The temperature was kept close to the original habitat conditions, with +1°C (day) and −1°C (night). The light regime was 12 h dark and 12 h light with an intensity of 100 to 150 μmol photons m^−2^ s^−1^, provided in a Percival LT‐36VL chamber (CLF Plant Climatics, Wertingen, Germany). Illumination for both the stock culture and exposure assays was provided by Narva BioVital 958 “full spectrum” fluorescence tubes, which also emit some UVA radiation (Figure S3 online). After 14 days, the exposed cells were harvested by centrifugation (500 × g, 10 min) and washed with acidified water (0.1 M HCl) to remove extracellular residual Fe. Subsequently, the material from the four treatments was used either for micro‐Raman spectroscopy or extraction by grinding the lyophilized cell pellet. The 5% (v/v) ethanolic aqueous extract was centrifuged (10,000 × g, 10 min), and the supernatant was used for colorimetric assays and HPLC.

### 2.3 Reference Phenols Complexation Assay

Complexation assays were performed using known phenolic standards. Iron complexes were prepared by combining iron(II) sulphate either with gallic acid in a 1:1 ratio, according to Lee et al. (2006), or with pure purpurogallin dissolved in dimethyl sulfoxide (DMSO) in this study. Furthermore, a complexation assay of 1,10‐phenanthroline with FeCl_2_ × 4H_2_O was performed in two solvents, deionised water and acetonitrile, according to Dalmieda et al. (2021). The molar ratio of phenanthroline and metal salt Fe(II) in the solution was 3:1, and the complex was characterised by Raman microscopy as a solid deposit after evaporation of the solvents. Subsequently, a comparison of their Raman spectra with those of the native purpurogallin‐glycoside of *A. alaskanum* from the field sample (WP274) collected at Morteratsch Glacier (isolated by HPLC fraction collection) was performed.

### 2.4 Confocal Raman Microscopy (Micro‐Raman)

For in vivo determination of the chemical composition of intracellular structures, confocal Raman microscopy was used (Moudříková, Sadowsky, et al. 2017; Moudříková, Nedbal, et al. 2017). The specimen preparation (low‐melting agarose immobilisation of the cells between a quartz microscopy slide and a coverslip) was carried out according to Barcytė et al. (2020). Two‐dimensional Raman maps were obtained with a WITec alpha300 RSA confocal Raman microscope (Oxford Instruments, WITec, Germany) with laser excitations at 532 nm, 647 nm, and 785 nm and various powers according to particular needs. The excitation powers were measured at the focal plane and are indicated in the corresponding figures. For focusing the excitation beam and collecting the scattered light, a 60× water‐immersion UPlanSApo W objective, NA 1.2 (Olympus, Japan) was used. Using a scanning step of 200 nm in both directions (x‐y) and an integration time of 100 ms per voxel, a full Raman map of an *Ancylonema* cell was acquired within 15 min. In some cases, only selected cellular areas were scanned, or single‐point spectra were measured at selected points. In that case, the Raman acquisition took only a few 10 of seconds or a few minutes.

In the case of measuring compounds that do not benefit from the resonance Raman effect, prior to Raman mapping, it was necessary to suppress the fluorescent background of chlorophylls by a low‐power wide‐area photobleaching, as described in Moudříková et al. (2016). Raman chemical maps were constructed by multivariate decomposition of the baseline‐corrected spectra into the spectra of pure chemical components using WITec Project Plus 5.1 software: Cosmic Ray Removal, Spectra Crop (cropping the end spectra portions that are burdened by detector errors), Graph Background Subtraction, True Component Analysis (TCA, Raman map decomposition into their individual components, whose signal was then averaged to obtain a final spectrum with the optimal ratio signal to stochastic noise). Single‐shot Raman spectra from the selected points in the cells were analysed according to the same protocol except for the TCA.

### 2.5 Colorimetric Fe‐Assay

The content of soluble iron was measured spectrophotometrically using 1,10‐phenantroline (5.0 g·L^−1^) with 0.2 M sodium acetate at pH 5.8 according to Harvey Jr. et al. (1955). Ammonium iron(II) sulphate was used for calibration in the concentration range of 0.5 to 5 ppm. A 200 μL sample extract was added to 1500 μL phenanthroline solution and mixed. After 48 h exposure in the dark at room temperature, the colorimetric assay was performed in an Agilent Cary 60 spectrophotometer at 508 nm.

### 2.6 Phenol's High‐Performance Liquid Chromatography (HPLC)

Algal phenols were extracted and chromatographically analysed and quantified according to Permann, Pierangelini, et al. (2022). Briefly, cells were lyophilised and ground with MTBE using mortar and pestle pre‐cooled with liquid nitrogen. Polar compounds were gained by phase separation of the MTBE against 5% ethanol. The latter aqueous phase was centrifuged and directly used for HPLC. The main peak in the chromatogram at 280 nm, which was identified earlier with LC/MS and NMR by Remias, Schwaiger, et al. (2012) as purpurogallin carboxylic acid‐6‐*O*‐β‐d‐glucopyranoside, was isolated via an Agilent 1200 analytical fraction collector and pooled from numerous injections. The collected fraction (which additionally contained the mobile phases water and acetonitrile with formic acid) appeared yellowish and was directly used for Raman measurements. Integrated peak areas of the chromatogram were quantified in gallic acid equivalents (GA eq).

## 3 Results

### 3.1 Field Sample Measurements

**FIGURE 1:**
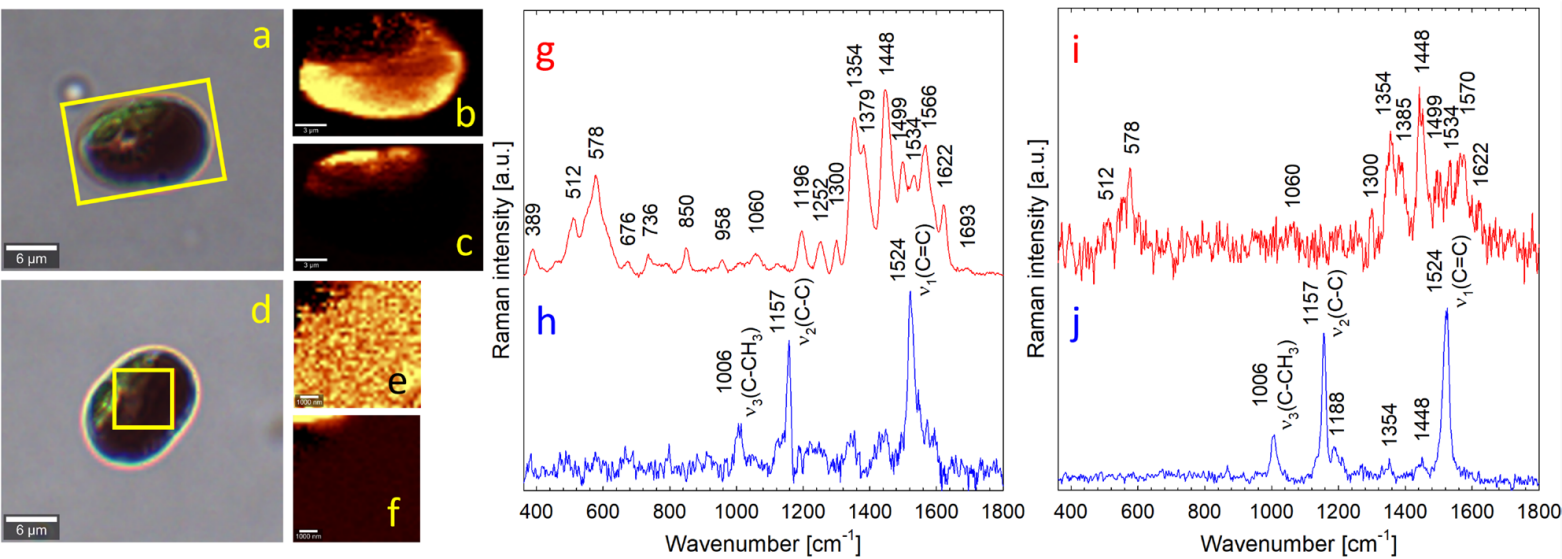
Bright‐field images of two *A. alaskanum* cells from the glacier obtained in vivo (a, d), Raman spectra of putative phenols (g, i) and those of carotenoids (h, j) as the most common cellular components detected in the scanned areas (yellow rectangle/square) by micro‐Raman. The corresponding chemical maps show the intracellular distribution of putative phenols (b, e) and carotenoid pigments (c, f). Raman spectra (g–j) are shown normalised to their respective maxima, vertically shifted and with marked band positions for better orientation. Chemical maps show that phenols are located in the dark vacuoles (b, e) and carotenoids in chloroplasts (c, f), the latter distinguishable as green areas in the bright‐field images (a, d). Raman maps were acquired using the “green” excitation at 532 nm with two different powers: 1.0 mW in (a) and 0.1 mW in (d). The higher excitation power caused photobleaching of carotenoids but allowed to obtain a spectrum of phenols with a better signal‐to‐noise ratio. On the other hand, due to less photobleaching, one order of magnitude lower excitation power allowed to obtain a less noisy spectrum of carotenoids. In both cases, the respective spectra were identical, differing only in the signal‐to‐noise ratio.

The dark vacuoles of cells of *A. alaskanum* harvested from the glacier were studied by confocal Raman microscopy (micro‐Raman). The chemical maps (Figure 1b,e) obtained with a 532 nm, low‐power excitation (0.1 and 1.0 mW) revealed a relatively homogeneous distribution of compounds providing a surprisingly intense cluster of characteristic Raman bands at about 1379, 1448, 1499, and 1566 cm^−1^ (Figure 1g,i). The high intensity of Raman scattering at such a low excitation power may be a consequence of resonance enhancement if the excitation wavelength falls within the absorption region of the chromophore but generally indicates the presence of condensed aromatic rings, the feature characteristic also of more complex phenols. The dark vacuoles of the field cells exhibited in vivo very similar Raman spectra, regardless of which of the three excitation lasers was used (Figure S4 online). In all three cases, high‐quality Raman spectra of the dark vacuole could be acquired with low excitation powers (even less than 1.0 mW). In contrast, the chemical maps of green chloroplasts showed prominent Raman bands at ca 1006, 1157, and 1524 cm^−1^ (Figure 1h,j), which are typical Raman markers of carotenoids present in algal plastids.

### 3.2 Complexation Assays With Reference Compounds

**FIGURE 2:**
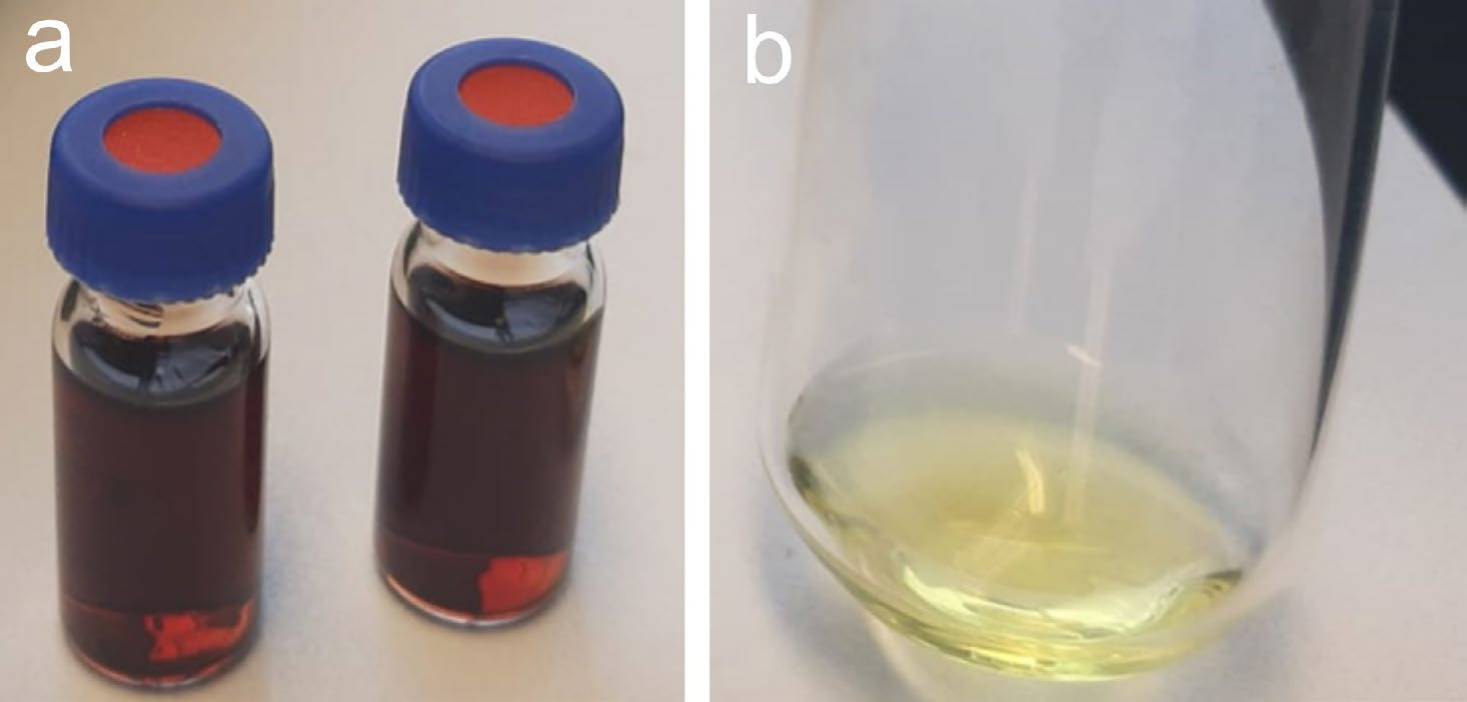
Visual comparison of the qualitative discrepancy in colour between *A. alaskanum* aqueous extracts from glacier samples (a, brownish) and the isolated main chromatographical peak (HPLC fraction collection) (b, yellowish).

**FIGURE 3:**
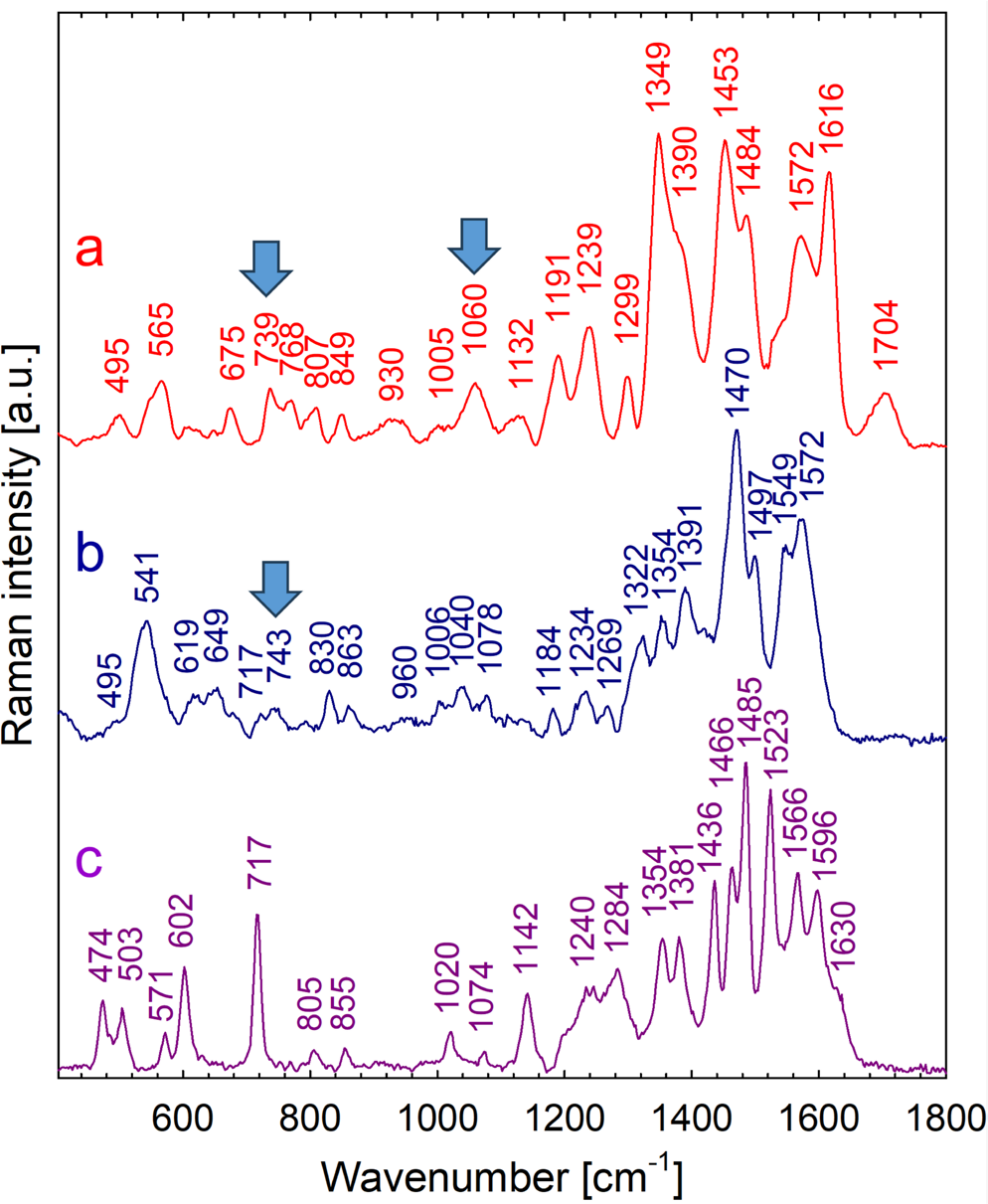
Comparison of Raman spectra of the isolated main chromatographic peak from *A. alaskanum* field cells, purpurogallin carboxylic acid‐6‐*O*‐β‐d‐glucopyranoside (a), Fe(II)‐purpurogallin complexes (b) and pure purpurogallin (c). Drops of the peak isolated by LC/FC, the DMSO solutions of Fe(II)‐purpurogallin, and the purpurogallin reference were dried on the quartz slides prior to measurement. The arrows indicate putative markers of iron complexes. Excitation: 532 nm, power 2.0 mW (a) and 0.5 mW (b, c).

**FIGURE 4:**
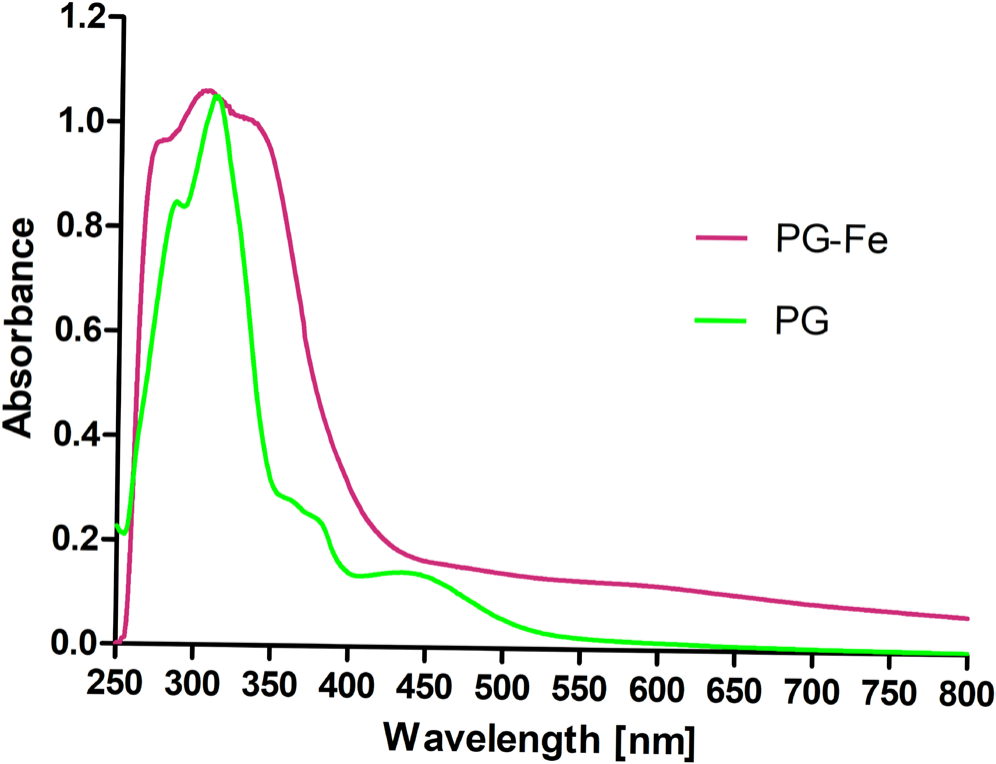
Comparison of the spectral absorbance between the purpurogallin standard (PG, green) and condensed iron‐purpurogallin (PG‐Fe, red), both dissolved in water. Note: Widening of the spectral absorption range of PG‐Fe compared to PG.

While aqueous extracts of *Ancylonema* field cells were dark brownish (Figure 2a), the main chromatographically isolated compound (purpurogallin glycoside) appeared yellowish (Figure 2b). To investigate the reason for this discrepancy in colour, a preliminary experiment was performed using Raman scattering (RS) with three phenolic reference compounds (gallic acid, purpurogallin, and phenanthroline). These compounds were subsequently subjected to iron complexation assays in vitro. Upon mixing with Fe, the gallic acid solution showed a rapid change in colour and in Raman spectra (Figures S5 and S6). This also applies to purpurogallin and phenanthroline. Raman spectra of purpurogallin exhibited several spectral changes after complexation (Figure 3b,c), reflecting the formation of bonds between purpurogallin and iron. For example, in the case of purpurogallin, there was a significant decrease in the relative intensity of the peak at 717 cm^−1^, and a new peak was observed at about 741 cm^−1^. The rapid change in colour and difference in spectral absorbance between solutions of the reddish purpurogallin standard and dark Fe‐purpurogallin is shown in Figure 4 and Figure S7 online. Finally, a rapid change in colour was also observed when comparing the solution of visually transparent 1,10‐phenanthroline standard and reddish Fe(II)‐1,10 phenanthroline complexes (Figure S8 online). Raman spectra of pure 1,10‐phenanthroline and of its complex with Fe(II) prepared in two polar solvents (deionised water and acetonitrile) are shown in Figure S9 online. From the comparison, it is clear that the solvent nature had no effect on the complex formation (Figure S9). Upon complexation with iron, several spectral changes were observed, in particular, the upshift of the band from 712 to 717 cm^−1^ to around 738–740 cm^−1^, which also seems to be characteristic of iron complexation with phenolic compounds. In the next step, Raman spectra of dried eSFM algae extracts alone and their complexes with 1,10‐phenanthroline were also recorded (Figure S10 online). The Raman spectrum of the extract mixture with phenanthroline is dominated by Raman markers of Fe(II)‐phenanthroline (Figure S11). The complexation of phenanthroline with iron cations present in eSFM algae extract (Figure S10) is evidenced by the Raman bands located at 558, 737, 877, 1059, 1211, 1454, and 1630 cm^−1^. This interpretation is based on a comparison with Figure S9.

To sum up, the overall Raman spectra of the three standards exhibited distinct differences. However, they notably shared the bands corresponding to putative phenol–iron complexes with the chromatographically isolated yellowish compound from *A. alaskanum* . Specifically, the peak at around 736–741 cm^−1^ was common between the algal isolate (Figure 3a), the iron‐purpurogallin (Figure 3b), and the iron‐phenanthroline complex (Figures S9 and S10). Additionally, the peak at around 1060 cm^−1^ was shared between the isolate and Fe(II)‐phenanthroline. Furthermore, Raman markers of Fe‐complexes were noticed in Raman spectra of the extract alone from eSFM cells when compared to the mixture of eSFM and Fe‐phenanthroline (Figure S10).

### 3.3 Exposure Experiment With the Lab Strain

**FIGURE 5:**
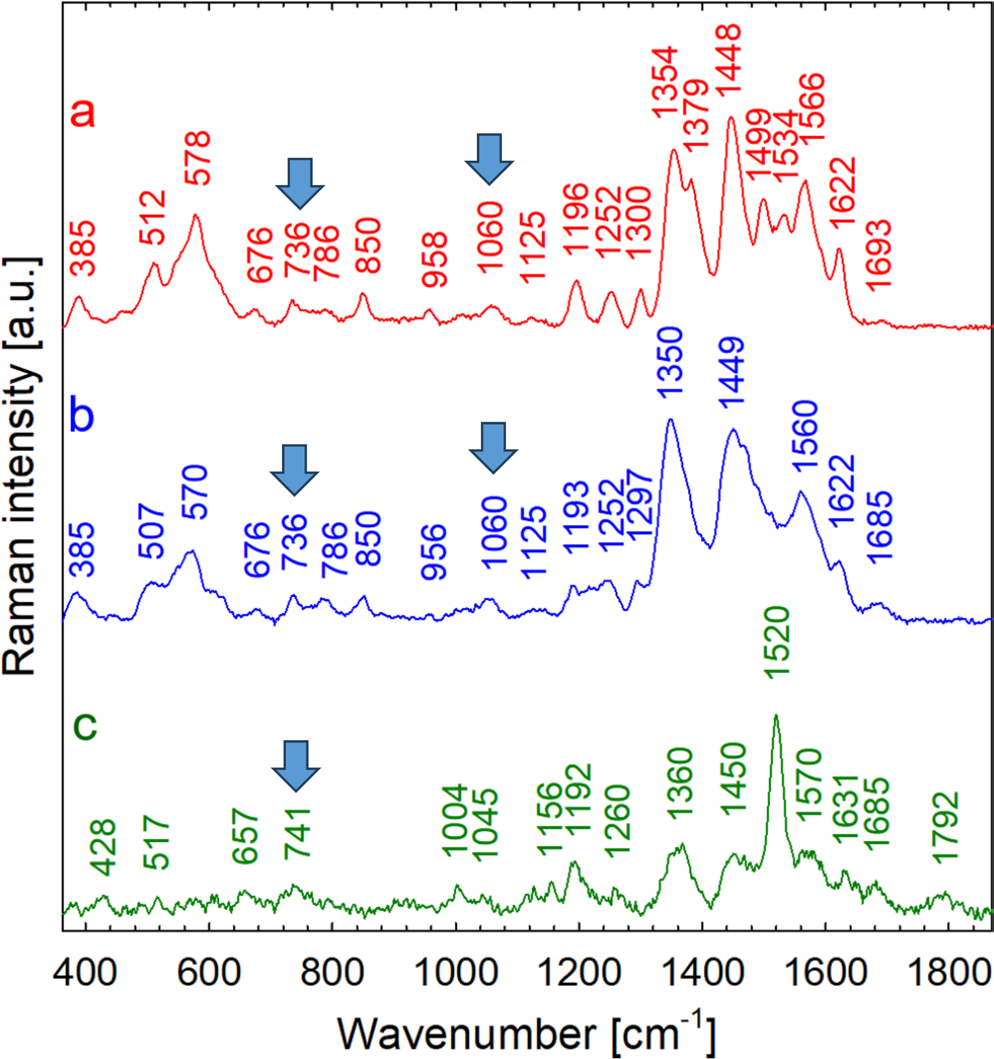
The Raman spectra of *A. alaskanum* , compared between dark vacuoles of field cells acquired in vivo (a), a dried sample prepared from the aqueous extract of stressed strain cells (depleted SFM without N and P; b) and visually unpigmented vacuoles of unstressed strain cells in vivo (grown in eSFM; c). The arrows indicate putative markers of iron complexes. Excitation: 532 nm, power 0.5 mW (a), 2.0 mW (b) and 1.0 mW (c).

**FIGURE 6:**
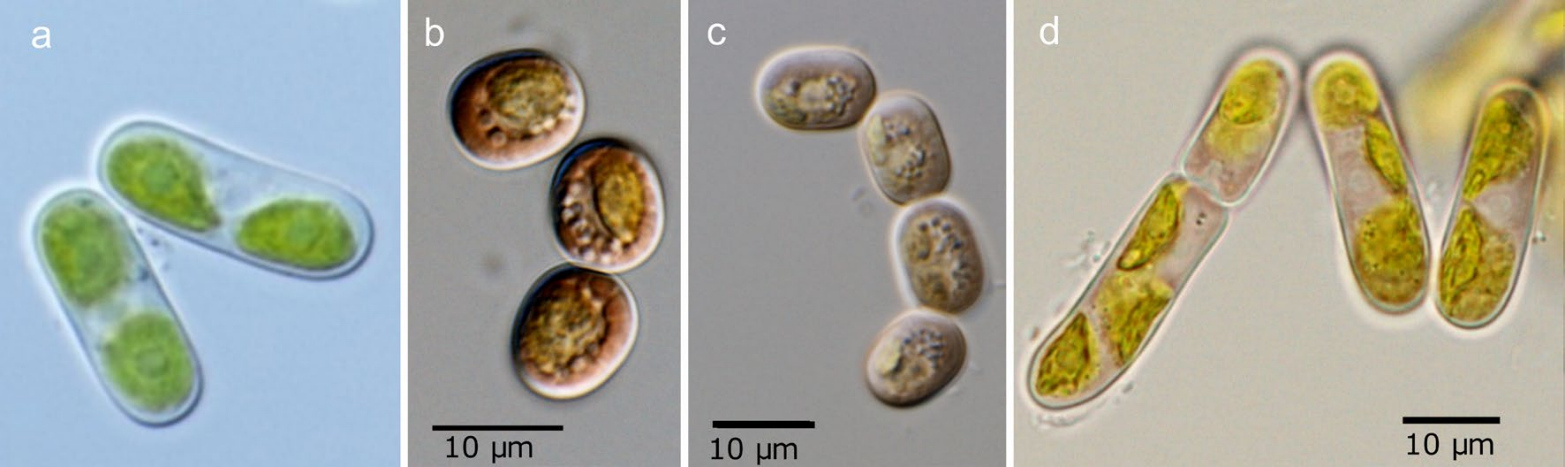
Light microphotographs of *A. alaskanum* (strain CCCryo 565–23), taken 14 days after exposure in four different media: growth medium (a, eSFM), depleted SFM (b, without N and P), deionised water (c) and depleted SFM with NaCl (d).

A strain of *A. alaskanum* (CCCryo 565–23) was used for testing whether the alga is able to form iron‐phenol complexes after cultivation under specific laboratory conditions. Under control conditions, iron was available in the Synthetic Freshwater Medium (eSFM) as FeCl_3_. The cells remained visibly green after 14 days of cultivation, and their vacuoles appeared unpigmented in light microphotographs (Figure 6a). Still, High‐Performance Liquid Chromatography (HPLC), and to a lower extent also micro‐Raman, confirmed the presence of phenols (compare Figure 5a,c). Furthermore, the main chromatographic peak and retention time were characteristic of purpurogallin (Figure S12 online). As under the control conditions, the stressed cells were harvested and extracted after 2 weeks of incubation, and their aqueous extracts analysed by HPLC and a colourimetric assay. The individual pigmentations of the algae extracts are shown in Figure S13 (online). Moreover, light microphotographs of the cells indicated that a slight brownish vacuolar pigmentation developed in the course of all three stress treatments (Figure 6), yet at a visually much lesser extent compared to the cells freshly collected from glaciers (Figure 1). Most importantly, the extracts of the stressed cells (SFM without N and P, and also with addition of NaCl, and in deionised water) exhibited Raman spectra closely resembling those of the dark vacuoles of the field‐collected ones (compare Figure 5a,b, for treatment with addition of NaCl and deionised water—not shown). In the case of the control cultivation, a certain amount of iron complexes was probably also detected by micro‐Raman (compare Figure 5a,c, presence of Raman bands at approximately 1192, 1360, 1450, and 1570 cm^−1^), but uncomplexed phenols probably prevailed in the extract, since the spectrum was dominated by the Raman band at 1520 cm^−1^, which should disappear with more extensive complexation.

Table 1 shows the mean content of total soluble phenols and iron in *A. alaskanum* after 14 days of exposure in all four media. In deionised water without any Fe‐source, the total phenol and iron content were the lowest. In the N‐ and P‐depleted medium, the median cellular phenol content was nearly identical to that observed under the control conditions (*p* > 0.05, Mann–Whitney test, Figure S14 online) but the iron content was significantly higher (*p* < 0.05, Mann–Whitney test, Figure S15 online).

**TABLE 1:** Content of total soluble phenolics and iron in aqueous extracts of *A. alaskanum* (strain CCCryo 565–23), extracted after 14 days of exposure in the control (eSFM) and three stress media (deionised water, SFM nutrient depleted, SFM nutrient depleted + NaCl).

Medium	GA equation TM^−1^ (mg·g^−1^)	Fe TM^−1^ (μg·mg^−1^)
Reference (eSFM)	48.1 ± 15.7	2806 ± 0.56
Deionised water	40.0 ± 7.3	1.213 ± 0.39
SMF nutrient depleted (no N, P)	43.0 ± 7.1	3.514 ± 0.41
SMF nutrient depleted (no N, P) + NaCl	32.5 ± 3.4	N/a

*Note:* The amount of phenolics is expressed in gallic acid equivalents (GA eq) per total dry matter (HPLC, *n* = 6, for SFM‐N‐*P* + NaCl—*n* = 3) and the soluble iron content per total dry matter (phenanthroline‐assay, *n* = 6, for deionised water *n* = 3).

## 4 Discussion

### 4.1 The Dynamic Relationship Between Iron, Pigmentation, and Environmental Stress in *Ancylonema*

Our results suggest that zygnematophycean glacier ice algae actively produce polyphenols, some of which are complexed with iron, and that the extent of their pigment production can be influenced by abiotic factors such as nutrient availability. Our experiments also showed that osmotic stress induced by the addition of NaCl to the medium can have a similar effect, increasing secondary pigmentation (Figure 6d). The dark pigmentation of *A. alaskanum* cultivated in a virtually Fe‐free medium (deionised water), where the active uptake of extracellular iron was limited, suggests the existence of intracellular iron deposits from earlier periods to be mobilised and redirected towards purpurogallin complexation, depending on environmental triggers. For instance, Zielińska‐Dawidziak (2015) demonstrated a highly dynamic and active role of ferritin iron complexes in the regulation of iron homeostasis, both in simple unicellular organisms and in complex eukaryotes.

An overview of microalgal iron uptake, short‐ and long‐term storage, and modulation of Fe requirements under stress conditions was recently provided by Lampe et al. (2024). As for glacier ice algae, the genes and processes related to iron acquisition or utilisation, which are known from other organisms, require further investigation. A comparative genomic analysis of Streptophyta evolution showed that one of the expanded gene families of *A. nordenskioldii* is associated with magnesium ion homeostasis (Bowles et al. 2024). Indeed, magnesium, which serves as a cofactor for some proteins, has a broad effect on the uptake and distribution of other essential elements like iron (Ahmed et al. 2023; Rolić et al. 2024). We propose that the function and place of the phenol and iron complexation in glacier ice algae are associated with protection against excessive irradiation, as earlier proposed by Williamson et al. (2020).

Regarding the biosynthesis of the phenolic iron complexes, Bowles et al. (2024) mapped the pathway for purpurogallin synthesis in *A. nordenskioeldii* through genome surveying but reported no association with enzyme‐driven iron complexation. Generally, iron sequestration from the environment by active secretion of phenols from roots is a well‐known phenomenon in land plants (Jin et al. 2007). However, it seems unlikely that vacuolic purpurogallin plays a similar role in glacier ice algae. Likewise, Doting et al. (2024) recently found no traces of phenols in the exometabolome (extracellular space) of an *Ancylonema* spp. bloom on the Greenland ice sheet, which points against secretion for enhancing iron uptake. Interestingly, this zygnematophycean secondary pigmentation is not exclusive or necessarily associated with the low temperatures of the ice habitat, as Busch et al. (2024) recently described a closely related mesophilic species from moorlands, *A. palustre* , which also shows prominent, similarly tinted vacuoles.

### 4.2 Adaptive Strategies of *Ancylonema* for Iron Sequestration in Glacier Ecosystems

In iron‐deficient ecosystems of the Earth, some algae have developed strategies to cope with limited access to iron as a micronutrient, for example substituting ferredoxin with flavodoxin, reducing the iron quota by 6% (Davidi et al. 2023). However, we suppose that *Ancylonema* sequesters more iron from its glacier habitat than is required for metabolism, storing it in vacuoles to mitigate potential intracellular toxicity (Sharma et al. 2016). Glacier meltwater is a rich source of bioavailable iron (II), and laboratory tests confirmed that a diatom grows more rapidly in media with input of glaciogenic particulates when compared to media supplemented with Fe from non‐glaciogenic dust sources (Shoenfelt et al. 2017). There are very few measurements of iron on glacier surfaces (Li et al. 2019) showing that the release of dissolved Fe (potentially labile) from melting Asian glaciers was 4–10 times higher than from the Greenland ice sheets or Antarctic ice sheet (Table 3 in Li et al. 2019). Future research shall bring more iron measurements on glaciers, also for insoluble iron (iron oxides) which influences the radiative properties of mineral dust and has an impact on ice melting processes: Many free iron oxides (ratio of free to total iron was 0.3–0.7) were found in cryoconite holes in Tibet (Cong et al. 2018), highlighting the need to focus on iron in the form of oxides rather than total iron measurements. Chemical characterisation of the glacier ice algae habitats indicated highly oligotrophic conditions in surface ice in both polar and alpine regions (Williamson et al. 2021; Millar et al. 2024). The melting of snow triggers a surge of nutrient release, including sulphate (SO_4_^2−^), ammonium (NH_4_^+^), nitrate (NO_3_^−^), calcium (Ca^2+^), chloride (Cl^−^), and sodium (Na^+^) (Kuhn 2001). Bioavailable nitrogen, predominantly in the form of ammonia, can be sequestered and mineralised in the sediment of cryoconite holes (Wynn et al. 2007). However, the remaining nutrients released from melting snowpacks are likely transported away from the surface ice environment through supraglacial meltwater rivers. Adaptation to such low nutrient conditions in surface ice can be seen in lower cellular macro‐nutrient content and elevated C:N and C:P ratios of glacier ice algal assemblages reported from Greenland (C:N:P of 1997:73:1) by Williamson et al. (2021) and from the Swiss glacier of this study (C:N in average 12.28 ± 0.70) (Millar et al. 2024). This is a significant deviation from classic Redfield stoichiometry (C:N:P = 106:16:1) (Redfield 1934). Under laboratory conditions, we demonstrated that iron complexation of purpurogallin was enhanced during stress exposure, while the level of phenol accumulation remained the same. We hypothesise that under such nutrient‐limited conditions, more Fe is stored in the vacuoles, which in turn generates more phenol‐Fe complexes.

### 4.3 Detection of Carotenoids Within *Ancylonema* Cells Using In Vivo Raman Microscopy

As shown in Figure 1, strong Raman bands at 1006, 1157, and 1524 cm^−1^ were detected in *A. alaskanum* field cells in areas identified as chloroplasts by light microscopy. These bands have been assigned to stretching vibration *v*_3_ (C—CH_3_), *v*_2_ (C—C), and *v*_1_ (C=C) (Němečková et al. 2021) and are well‐known Raman markers for carotenoids. The carotenoid composition of *Ancylonema* was reported to contain lutein, ß‐carotene, violaxanthin, zeaxanthin, antheraxanthin, and neoxanthin (Williamson et al. 2020). Although carotenoids absorb light preferentially in the blue region of the visible spectrum, they can generate intense Raman scattering even outside their resonance region due to their unique properties. The long chain of conjugated double bonds in carotenoids contributes to their high polarizability and Raman cross‐section. These characteristics enable carotenoids to scatter radiation and emit a Raman signal with high probability and intensity, even when excited with wavelengths not within their specific resonance region. As a result, high‐quality Raman spectra of carotenoids in chloroplasts living cells can be obtained at virtually any excitation wavelength, including the “green” excitation at 532, the “red” excitation at 647, and “the far‐red” excitation at 785 nm. This is achievable with relatively low excitation powers and without causing significant photodamage to the photosynthetic apparatus (this study).

### 4.4 Low‐Power Raman Imaging of Phenols in Vivo in *Ancylonema*

Using very low powers (even less than 1.0 mW) across all three excitation wavelengths, Raman signals of carotenoids were detected alongside distinct Raman spectra of phenols, localised exclusively in the dark vacuoles. The fact that such low excitation powers were sufficient is probably related to the dark colour of the phenol–iron complexes absorbing throughout a wide spectral range and thus benefiting from the resonance enhancement of RS. The Raman spectrum of phenols differed significantly from the spectra reported for mitochondria, lipids, starch, nucleus, polyphosphates, cell wall, or any known compound detected by Raman microscopy in a streptophytic alga from the genus *Cylindrocystis* (Barcytė et al. 2020); the Raman detection of non‐phenolic compartments requires significantly higher excitation powers.

### 4.5 Intracellular Reservoirs of Phenolic Compounds

As can be seen from light microphotographs, the vast majority of *Ancylonema* cell volume is occupied by a central vacuole, as a typical feature for streptophytes. Moreover, the phenols could be present in small (< 2 μm), spherical, electron‐dense bodies that have been reported near the chloroplast in *Mougeotia* (Tretyn et al. 1992), *Zygogonium ericetorum* (Aigner et al. 2013) and at the periphery of *Zygnema* protoplasts (Holzinger et al. 2018). The related zygnematophycean alga *Zygogonium ericetorum* , known from acidic and extremely nutrient‐poor habitats such as high alpine streamlets or exposed mountainous peatbogs, has pinkish vacuoles (Herburger et al. 2016).

### 4.6 Complexation Assays of Phenolic Compound References

To test whether iron‐phenol complexes are responsible for the observed optical properties in *Ancylonema*, three reference compounds with known structures (gallic acid, purpurogallin, and 1,10 phenantroline) were used to form iron complexes, and their respective Raman spectra were compared with the Raman signal detected in the dark vacuole of *Ancylonema*.

Gallic acid is recognised as a precursor in purpurogallin synthesis (Bowles et al. 2024), and gallic acid moieties are widespread in Zygnematophyceae (e.g., Aigner et al. 2013; Han et al. 2012). Iron complexes of gallic acids constitute dark pigments and have been used as traditional components of ink (Ponce et al. 2016). Raman spectra of gallic acid complexed with iron(II) sulphate obtained in this study (Figure S6) correspond well to those previously shown in Lee et al. (2006). However, we found no evidence of this compound in *A. alaskanum* . Although the complexation capabilities of purpurogallin have not yet been reported, two or more molecules of purpurogallins can be assumed to complex with one iron atom, analogically to the iron‐quercetin 1:2 complexes (Kejík et al. 2021). Since, to the best of our knowledge, Raman spectra of purpurogallin (either alone or in complex with iron) have not yet been studied and published, there is no theoretical or phenomenological basis for interpreting our results. A potential analogy can be drawn from the complexes of other biologically important aromatic compounds with iron, which have already been studied using Raman spectroscopy. For example, phenanthroline complexes with iron have been investigated, and differences between the Raman spectra of free phenanthroline and iron‐phenanthroline complexes were previously described by Dalmieda et al. (2021), who identified characteristic Raman markers for iron complexation.

### 4.7 Spectroscopic and Chromatographic Evidence for Polyphenol‐Iron Complexation in *Ancylonema*

Purpurogallin‐glycosides comprised the main chromatographic peak in the aqueous extract of *Ancylonema* field cells (Remias, Schwaiger, et al. 2012; Procházková et al. 2021) and *Ancylonema* strain cells (this study). Since the yellowish fraction and iron‐purpurogallin complex shared only a few important Raman bands (Figure 3a,b), we hypothesise that this yellowish solution may have a more complex phenolic composition than previously reported. For the present complexation studies with iron, only pure purpurogallin was available as a reference compound. Assuming that non‐aromatic glucose moieties of purpurogallin‐glycosides themselves do not contribute significantly to the resonantly enhanced Raman signal, the dramatic spectral changes induced by the complexation of purpurogallin with iron were used for comparison with the Raman spectra of the chromatographic extracts from field cells. Certain spectral differences between the field‐cells extracts, vacuoles of fresh field cells, and the reference iron‐purpurogallin complex may also be due to the different states of the samples: on the one hand, solubilised form in vacuoles (Figure 1), on the other hand, dried precipitates in the solid phase (Figure 3). Based on the Raman spectra, we cannot exclude the possibility that other complexed and non‐complexed polyphenols are present in the isolated chromatographic peak (Figure 3a,c). However, even when using purpurogallin as the only available reference compound, it is evident that its complexation with iron causes dramatic changes in its Raman spectrum, for example, the elimination of the intense band at 717 cm^−1^, which is likely replaced by a significantly weaker band in the region of 739–743 cm^−1^ (compare Figure 3b and Figure 3c).

In general, different polyphenols possess multiple hydroxyl groups capable of chelating metal ions like iron (Fe^2+^ or Fe^3+^) in various ways. It is also known that their antioxidant properties are mainly induced by the iron chelation effect (Pan et al. 2022). When iron forms complexes with polyphenols, it may alter its solubility. In many cases, these complexes can enhance the solubility of iron in aqueous solutions. Accordingly, transmission electron microscopy of the field *A. alaskanum* cells showed prominent, homogenously electron‐dense vacuoles with electron‐translucent cytoplasmic strands, but no particles (crystals) within vacuoles (Remias, Holzinger, et al. 2012). Furthermore, the quantified soluble iron in the assay does not represent exclusively iron complexes but encompasses all forms of soluble iron. The remarkable colour change/difference (Figure 2) between the brownish raw aqueous extract from glacier cells and the yellowish colour of the isolated main compound may be due to a combination of the factors, on the one hand, including concentration and pH; on the other hand, due to chromatographic reasons, the isolated fraction with the main peak lacked large phenol–polymers, which likely dominate the overall dark pigmentation of the vacuoles (Figure S16 online). Due to the size of polyphenol–iron complexes, no LC/MS peaks were generated and therefore may not be reported earlier; nonetheless, these complexes remained detectable spectrophotometrically and by Raman spectroscopy.

The high spectral absorption of dark aqueous extracts from *Ancylonema* field cells in the UV range, along with broad absorption throughout the VIS range, was experimentally reproduced in this study also in vitro by the preparation of iron complexes with purpurogallin. It is highly probable that the screening effect of the iron–polyphenol complexes accumulated in dark vacuoles is vital for the survival of microalgae on exposed glacier surfaces (Williamson et al. 2020). This points to sophisticated photoprotection strategies in streptophytic green algae. Given the extent of glacier algal blooms in polar and alpine regions (Wang et al. 2018), the high amount of dissolved iron detected in cells of glacier ice algae may deeply influence concepts of iron budgets/cycling (Hawkings et al. 2014; Raiswell et al. 2018; Kappler et al. 2021; Krause et al. 2024).

## Author Contributions

**Lenka Procházková:** conceptualization, investigation, visualization, formal analysis, writing – review and editing, writing – original draft, resources. **Peter Mojzeš:** visualization, formal analysis, investigation, writing – review and editing. **Jan Ráček:** investigation, writing – review and editing. **Linda Nedbalová:** writing – review and editing, funding acquisition. **Daniel Remias:** writing – review and editing, writing – original draft, investigation, conceptualization, visualization, funding acquisition, resources.

## Conflicts of Interest

The authors declare no conflicts of interest.

## Supporting information


Data S1.

## Data Availability

All the data are provided in the manuscript and in the Supporting Information.
