# Supplementary material for: Phenolic Iron Complexes Protect Glacier Ice Algae (Zygnematophyceae) Against Excessive UV and VIS Irradiation
Source: Environ Microbiol Rep. 2025 Jul 14;17(4):e70149. doi: 10.1111/1758-2229.70149 (PMC12257149; doi:10.1111/1758-2229.70149)
Supplement: Supplementary file 1 — Data S1. [file EMI4-17-e70149-s001.pdf]

Supplementary information

Phenolic iron complexes protect glacier ice algae (Zygnematophyceae) against excessive UV and VIS irradiation

Lenka Procházková[1]*, Peter Mojzeš[2], Jan Ráček[3,4], Linda Nedbalová[1] and Daniel Remias[5]*

Affiliations

[1]Department of Ecology, Faculty of Science, Charles University, Viničná 7, 128 00 Prague, Czech Republic,

[2]Institute of Physics, Faculty of Mathematics and Physics, Charles University, Ke Karlovu 5, 121 16 Prague, Czech Republic,

[3]Department of Botany, Faculty of Science, Charles University, Benátská 2, 128 00 Prague, Czech Republic,

[4]Institute of Microbiology, Czech Academy of Sciences, Vídeňská 1083, 142 00 Prague, Czech Republic,

[5]Department of Environment & Biodiversity, Hellbrunnerstr. 34, University of Salzburg, 5020 Salzburg, Austria,

***Correspondence:**

Lenka Procházková

Tel: +420 723 109 482

e-mail: lenka.prochazkova@natur.cuni.cz

Daniel Remias

Tel: +43 662 8044 5554

e-mail: daniel.remias@plus.ac.at

Supplementary Table 1. Recipe for "stress medium" - Synthetic Freshwater Medium (SFM) with depleted nutrients (-N -P) and additional 1mM/NaCl.

**Stock #**	***Components***	***Stock solution***	***Add per L of SFM -N -P***
**1.**	HEPES puffer	238.10 g / L dH_2O	1 mL
**2.**	$MgSO_4 \times 7\ H_2O$	20.00 g / L dH_2O	2.5 mL
**3.**	Na_2CO_3	32.00 g / L dH_2O	0.6 mL
**4.**	NaCl	5.84 g / L dH_2O	10 mL
**5.**	H_3BO_3	1.00 g / L dH_2O	1 mL
	Trace Metal Solution:		1 mL
	Preparation of Trace Metal Solution		
	$Na_2EDTA \times 2\ H_2O$: 4.36 g		
	$FeCl_3 \times 6\ H_2O$: 3.15 g		
Dissolve in 1 L dH_2O, then add 1 mL of Prim. Trace Metals each:			
	Primary Trace Metals:		
	K_2CrO_4	0.194 g / 100 mL dH_2O	
	$CoCl_2 \times 6\ H_2O$	1.00 g / 100 mL dH_2O	
	$CuSO_4 \times 5\ H_2O$	0.25 g / 100 mL dH_2O	
	$MnCl_2 \times 4\ H_2O$	18.00 g / 100 mL dH_2O	
	$Na_2MoO_4 \times 2\ H_2O$	1.89 g / 100 mL dH_2O	
	$NiSO_4 \times 6\ H_2O$	0.27 g / 100 mL dH_2O	
	H_2SeO_3	0.13 g / 100 mL dH_2O	
	Na_3VO_4	0.184 g / 100 mL dH_2O	
	$ZnSO_4 \times 7\ H_2O$	2.20 g / 100 mL dH_2O	

Add stocks #1 to 5 in 950 mL dH_2O.
Adjust the pH to 6.0.
Bring to final volume 1 litre and autoclave.
Store Stock 1 dark and cool!

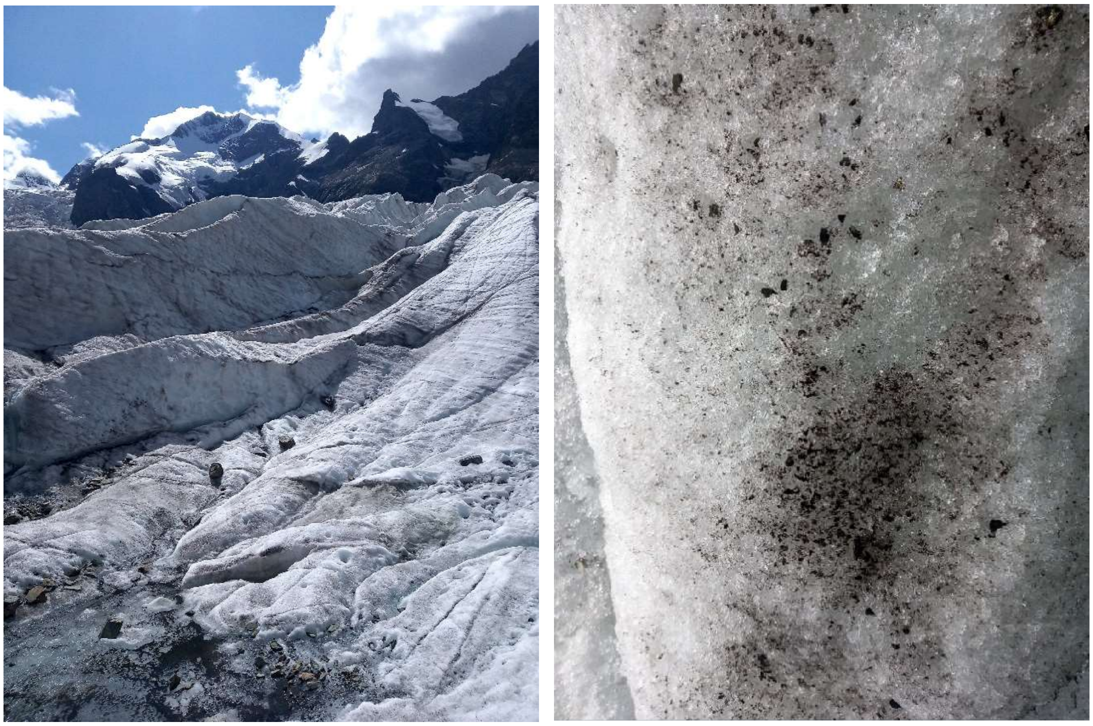

Supplementary Fig. 1. Field site of the glacier ice alga *A. alaskanum* (sample WP274). Morteratsch Glacier, Graubünden, Switzerland. (Left) overview with Piz Bernina (4048 m a.s.l.) in the background, (right) close-up view of cryoconite and algae-loaded ice surface.

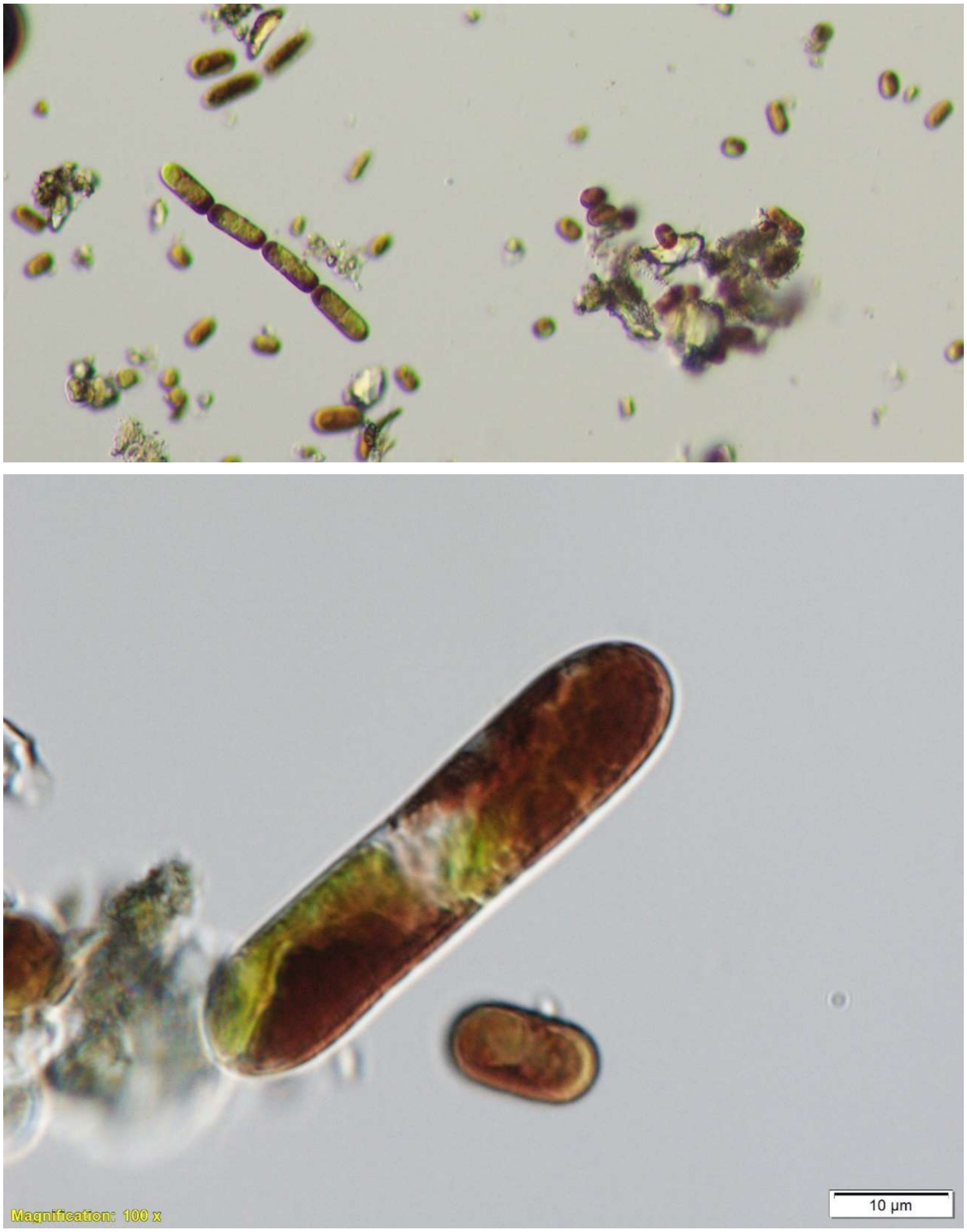

Supplementary Fig. 2. Field sample WP274, dominated by *A. alaskanum* (small cells, unicellular). Several single-cell to short filaments-forming species *A. nordenskioeldii* were also present. (Upper) overview (200× magnification) and (bottom) detail view of both species (1000× magnification). The green chloroplast is partly visible but largely masked by the brownish dark vacuoles in the cytoplasm.

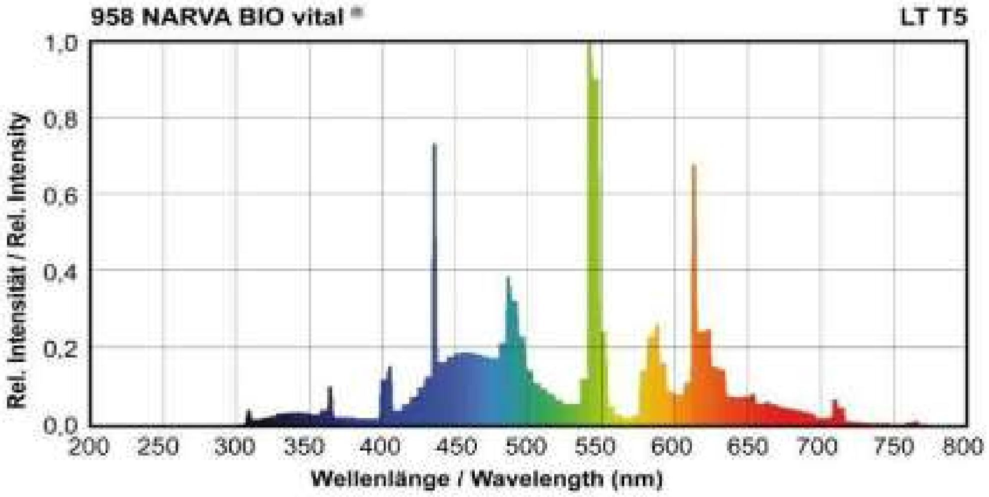

Supplementary Fig. 3. Spectral emittance of the Narva BioVital lamps used both for the stock culture and exposure assays.

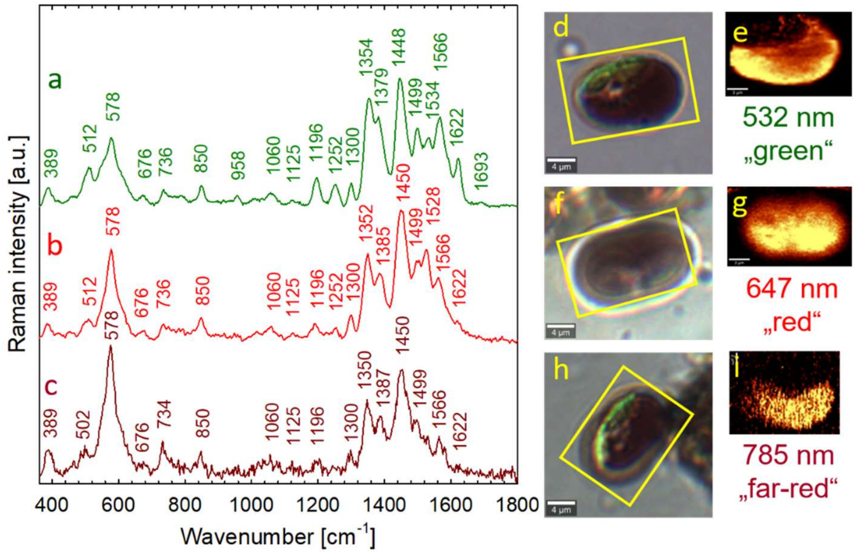

Supplementary Fig. 4. In order to find the suitable excitation wavelength for Raman spectra of the dark vacuole, *A. alaskanum* cells from field samples were tested *in vivo* (d, f and h): Comparison of Raman spectra (a, b, and c) and corresponding chemical maps (e, g, and i) obtained by three different excitations: "green" 532 nm, "red" 647 nm, and "far-red" 785 nm, respectively. In all three cases, the excitation power was 1.0 mW. The dark vacuoles of the field cells exhibited *in vivo* very similar Raman spectra, regardless which of the three excitation lasers was used

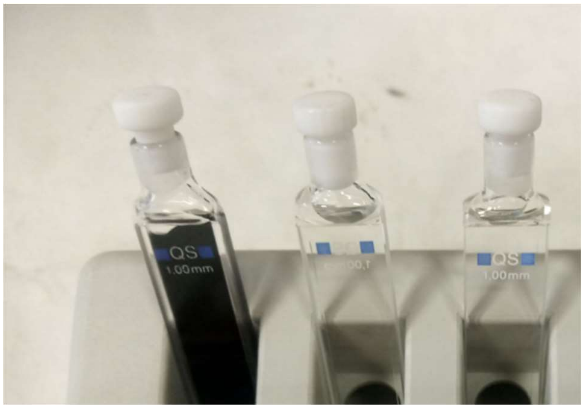

Supplementary Fig. 5. Visual comparison of solutions of gallic acid iron complexes (left), iron sulphate heptahydrate (middle) and gallic acid (right).

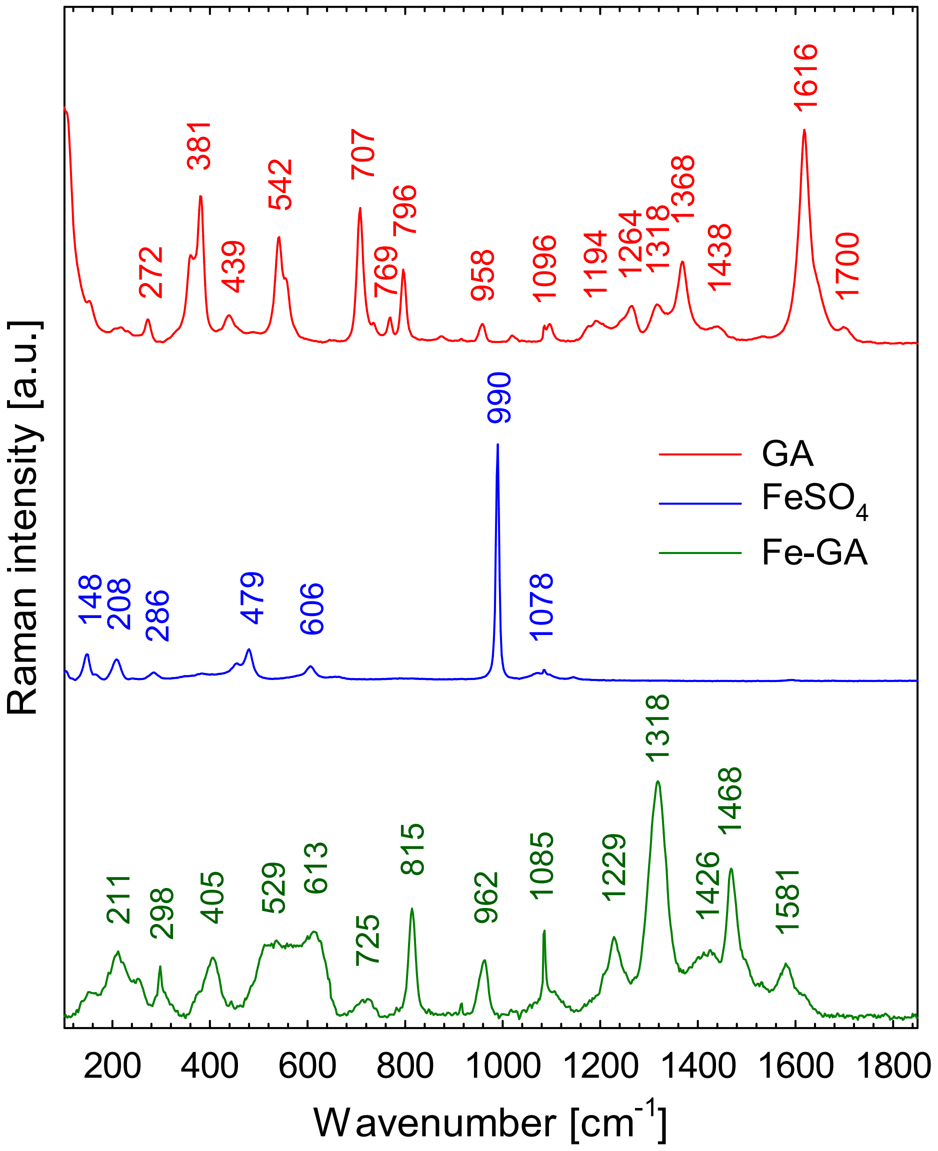

Supplementary Fig. 6. Comparison of Raman spectra of (upper) the free phenol gallic acid, (middle) the substrate iron (II) sulphate heptahydrate and (bottom) the resulting iron complex of gallic acid and $FeSO_4$. Far-red laser excitation (785 nm), 1 mW power. All chemicals were measured as dried deposits on a quartz slide.

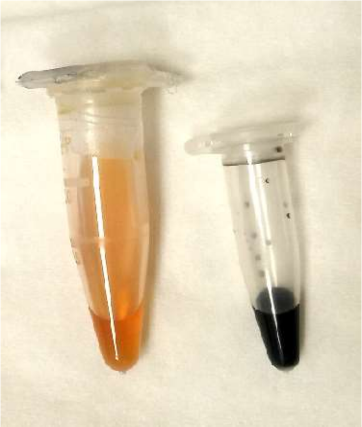

Supplementary Fig. 7. Visual comparison of solutions of purpurogallin (left) and iron-purpurogallin (right).

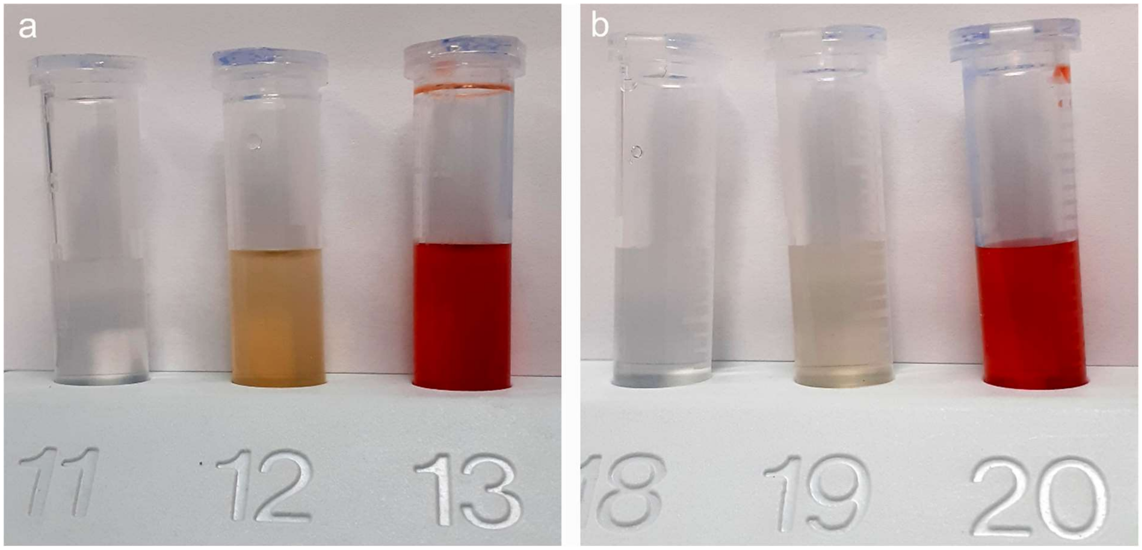

Supplementary Fig. 8. Visual comparison of solutions of 1,10-phenanthroline (positions 11 and 18), $FeCl_2 \times 4H_2O$ (positions 12 and 19), and Fe(II)-1,10-phenanthroline complexes (positions 13 and 20) when it was dissolved in (a) acetonitrile or (b) deionized water.

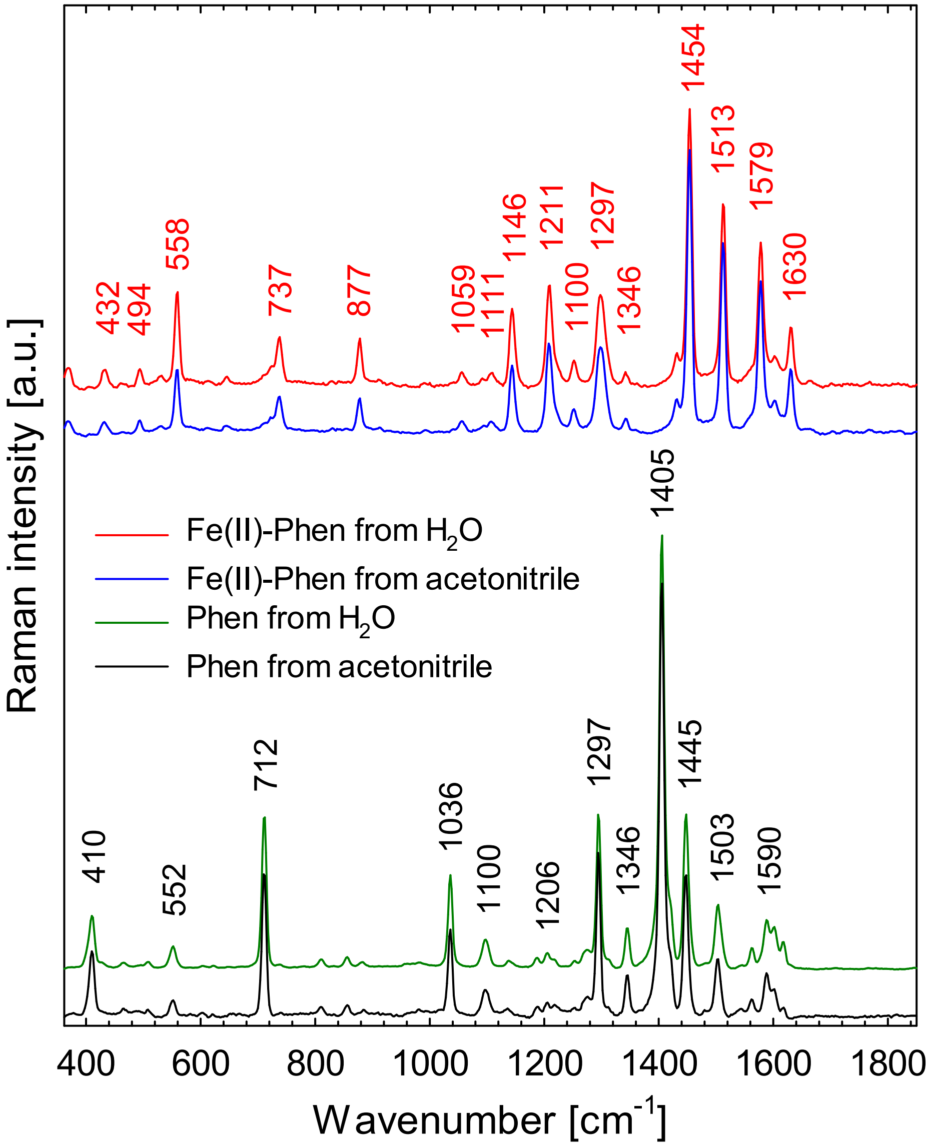

Supplementary Fig. 9. Comparison of Raman spectra of pure phenanthroline (Phen) and its complex with Fe(II) (Fe(II)-Phen) prepared according to the published procedure (Dalmieda *et al.* 2021). Both substances were measured as solids after evaporation of the solvent on a quartz slide. The Fe(II)-phenanthroline complex was prepared alternatively in deionized water and in acetonitrile. As can be seen from the comparison of the respective spectra, the solvent had no effect on the formation of the complex. Excitation: 532 nm, power 0.5 mW.

It is worth noting that although the same preparation procedure was followed as in Dalmieda *et al.* 2021, the spectra of both phenanthroline and Fe(II)-phenanthroline exhibit some differences when compared with those published by Dalmieda *et al.* 2021. The spectrum reported by Dalmieda *et al.* 2021 as the spectrum of the Fe(II)-phenanthroline complex seems to be, according to our observations, the spectrum of a mixture of pure phenanthroline and the Fe(II)-complex. The spectra reported here represent the spectra of the pure forms of both substances.

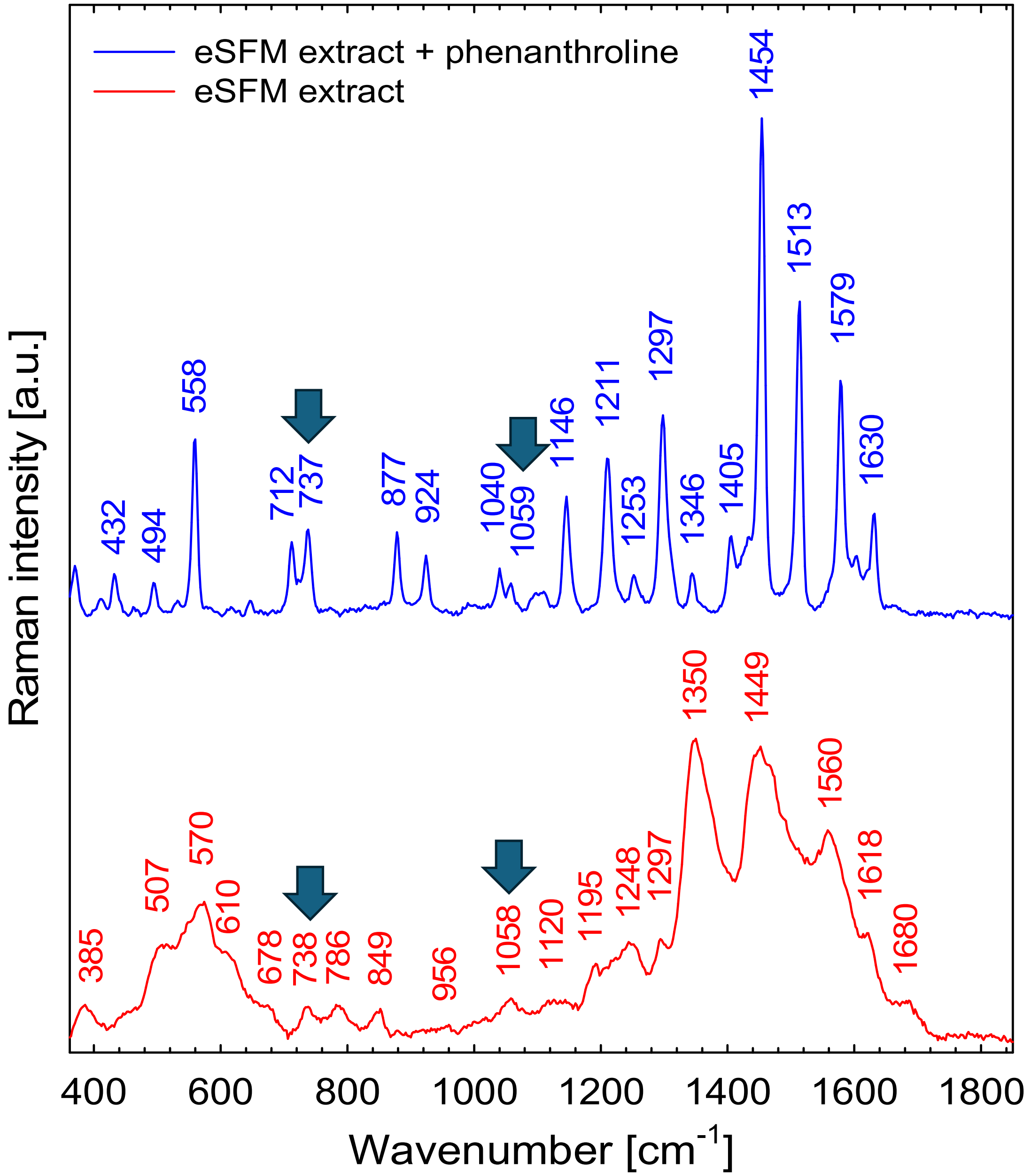

Supplementary Fig. 10. Comparison of Raman spectra of eSFM algae extract alone (red line) and eSFM algae extract mixed with phenanthroline (blue line). The Raman spectrum of the mixture is dominated by the spectral contribution of Fe-phenanthroline. The complexation of phenanthroline with iron is evidenced by the Raman markers characteristic for Fe-phenanthroline located at 712, 737, 1060 and 1211 cm^{-1} (compare Dalmieda *et al*. 2021). The arrows indicate putative markers of iron complexes found in eSFM algal extract. Excitation: 532 nm, power 0.5 mW (for eSFM extract with phenanthroline) and 2 mW (for eSFM extract only).

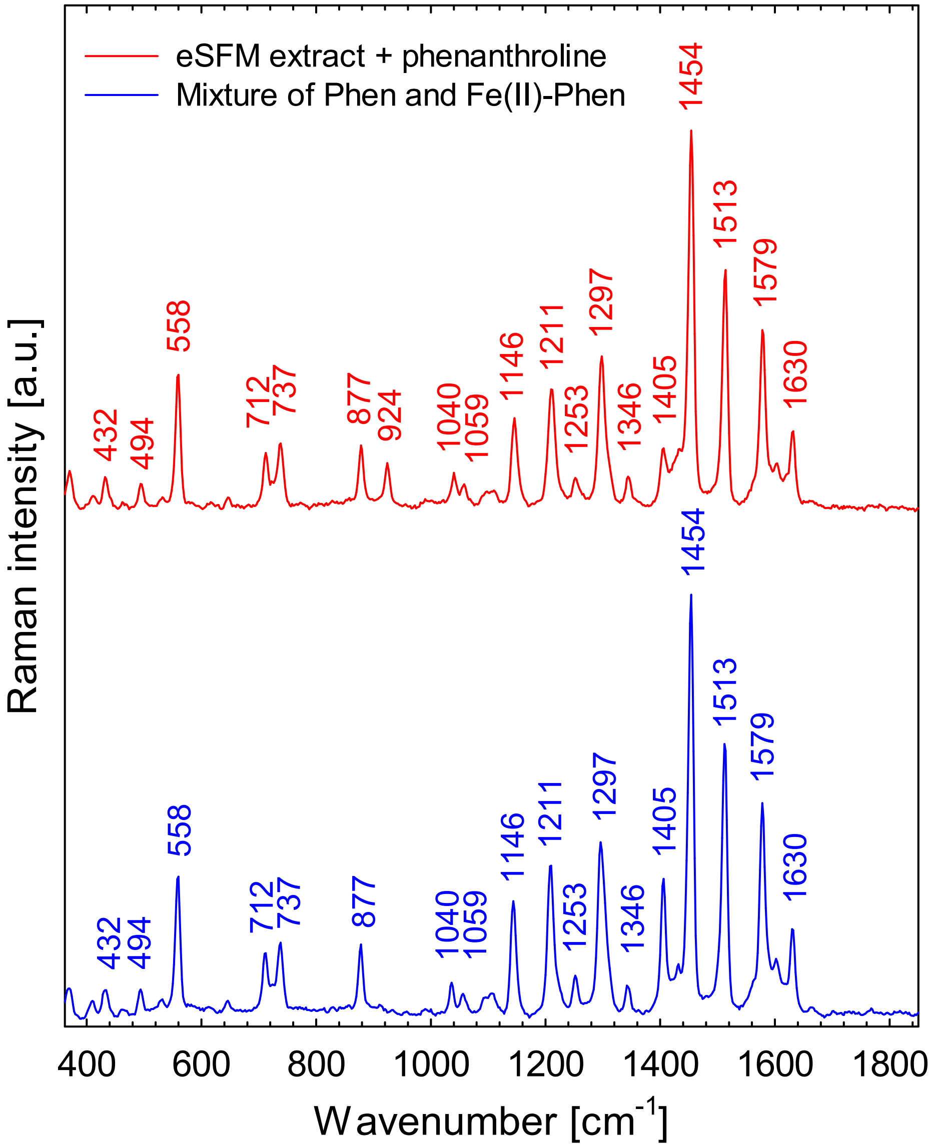

Supplementary Fig. 11. Comparison of Raman spectra of eSFM algae extract mixed with phenanthroline (red line) with a linear combination of spectra of pure phenanthroline and Fe(II)-phenanthroline complex (blue line). The very good agreement of the spectra supports the interpretation that after the addition of phenanthroline to the eSFM algae extract, phenanthroline was in excess and that only for a part of the added phenanthroline a sufficient amount of iron ions was available. Excitation: 532 nm, power 0.5 mW.

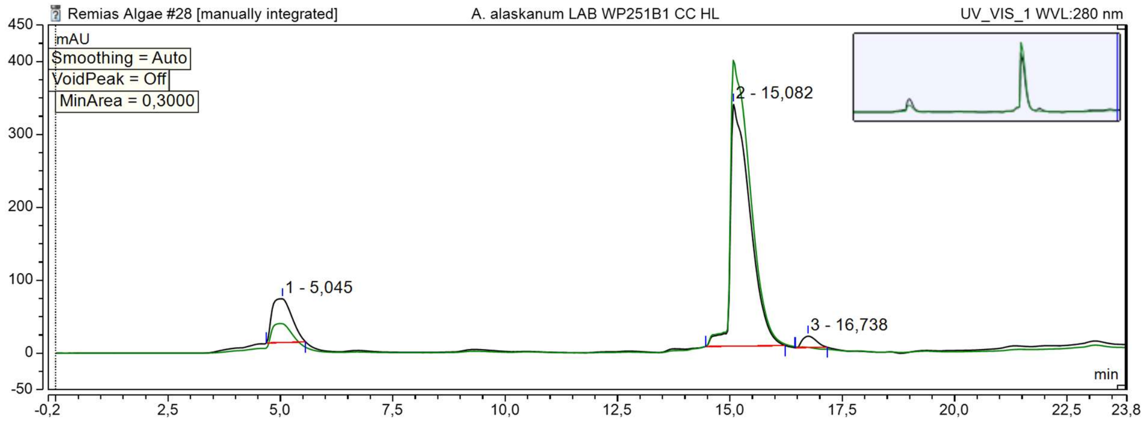

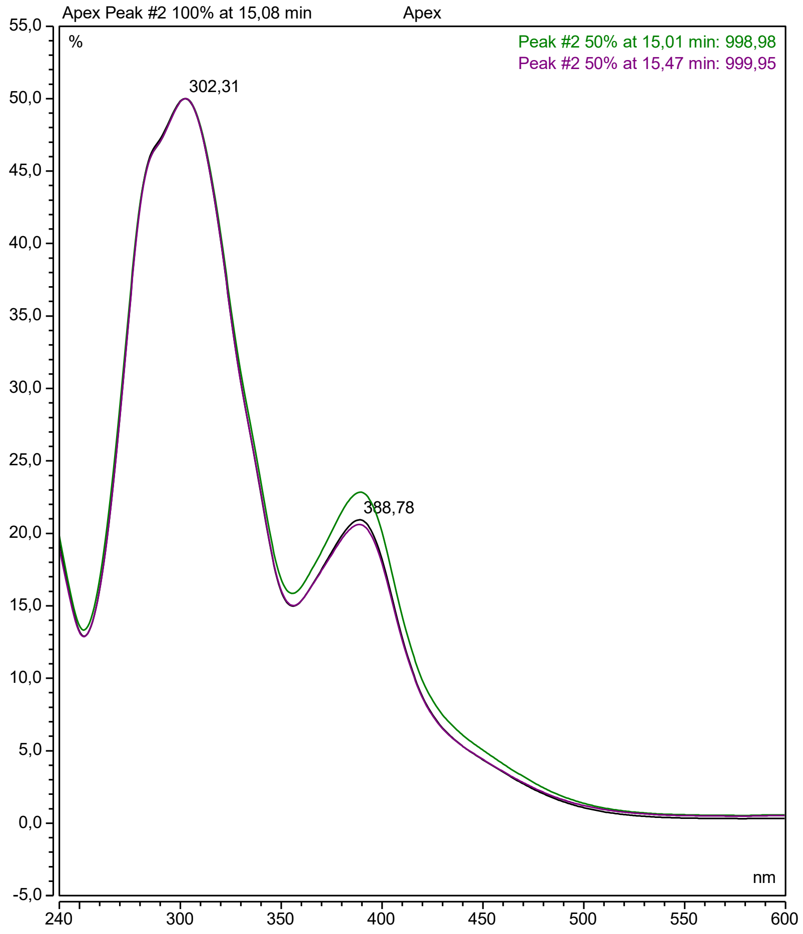

Supplementary Fig. 12. Chromatogram (@ 280 nm) of the aqueous (20% Ethanol) extract of *A. alaskanum* lab strain (CCCryo 565-23), extracted from green cells after cultivation for 14 days in eSFM (control conditions), acquired with a diode array detector. The main peaks at 15 min represents purpurogallin carboxylic acid-6-O-β-d-glucopyranoside (upper graph). The LC-online spectra (water/acetonitrile/formic acid) of these peaks show the characteristic absorption of the compound (lower graph).

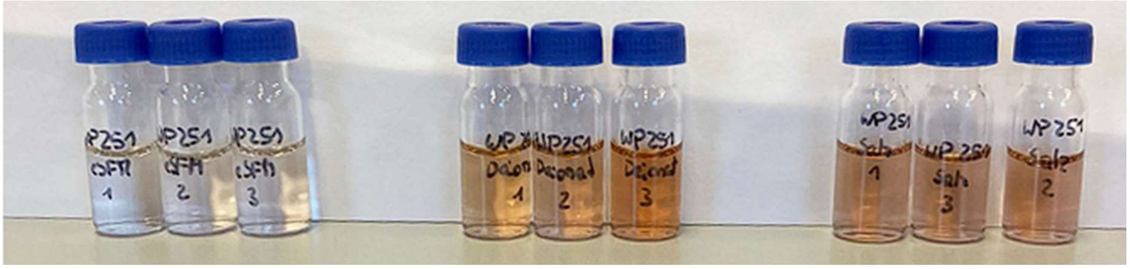

Supplementary Fig. 13. Visual comparison of aqueous (5% ethanolic) extracts of *A. alaskanum* (strain CCCryo 565-23) after 14 days exposure in three different media: Reference (eSFM, left), deionized water (middle), ”stress medium” (N- and P-depleted, salt addition, right).

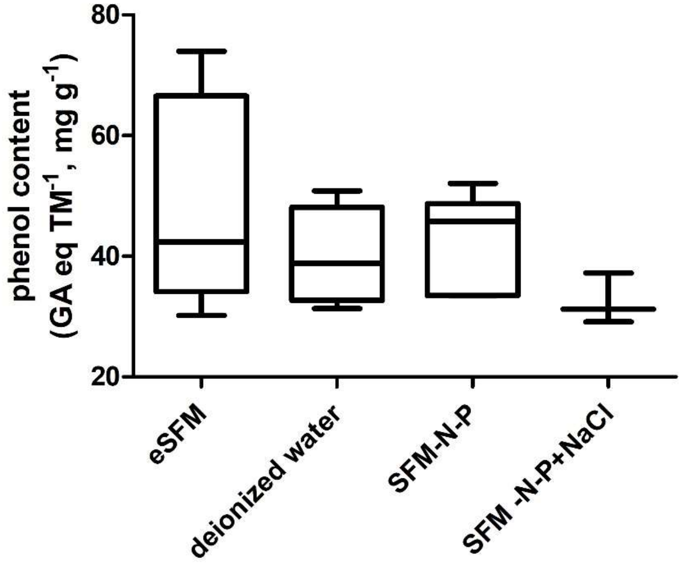

Supplementary Fig. 14. Phenol content of aqueous extract of *A. alaskanum* lab strain (CCCryo 565-23) in control conditions and three stressed media (n=6; for SFM -N-P +NaCl n=3). In the N- and P-depleted medium, the median cellular phenol content was nearly identical to that observed under the control conditions (p>0.05, Mann-Whitney test).

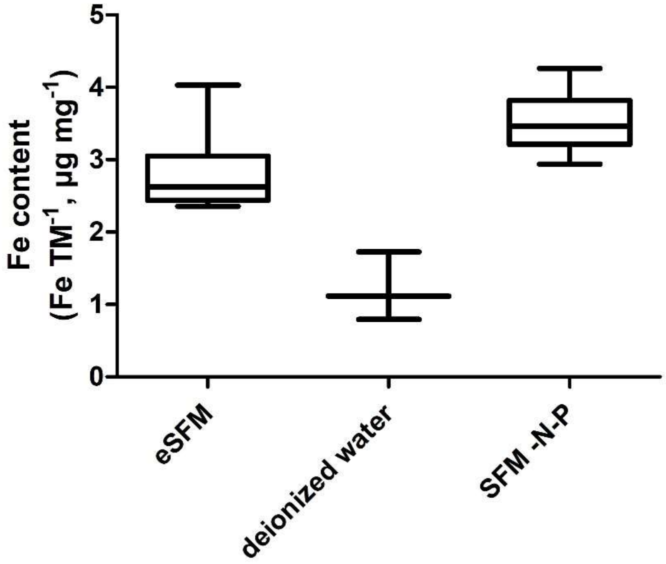

Supplementary Fig. 15. Fe content of aqueous extract of *A. alaskanum* lab strain (CCCryo 565-23) in control conditions (eSFM) and two stressed media (deionized water, n=3; SFM-N-P, n=6). For SFM -N-P +NaCl data is not available. In the N- and P-depleted medium, the median cellular iron content was significantly higher to that observed under the control conditions ($p<0.05$, Mann-Whitney test).

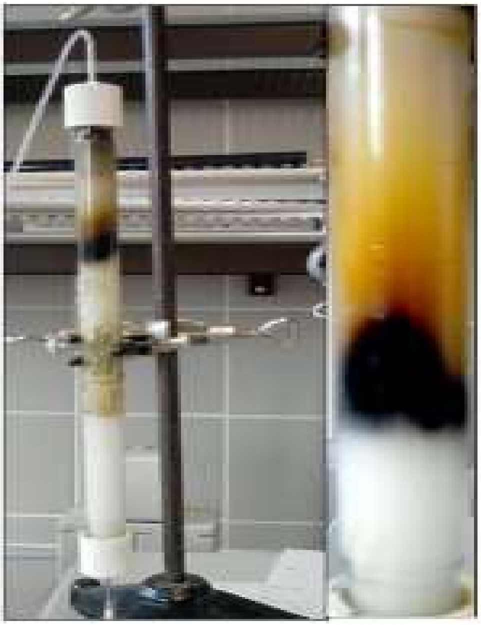

Supplementary Fig. 16. Flash-chromatography (stationary phase: Sephadex LH-20) of aqueous extracts of field samples of *A. alaskanum*. It shows a dark bulk of putative iron complexes, whereas the yellowish purpurogallin-glycoside has a different retention.
